# HLAsupE: an integrated database of HLA supertype-specific epitopes to aid in the development of vaccines with broad coverage of the human population

**DOI:** 10.1186/s12865-016-0156-x

**Published:** 2016-06-16

**Authors:** Shufeng Wang, Ling Guo, Dong Liu, Wei Liu, Yuzhang Wu

**Affiliations:** Institute of Immunology PLA, Third Military Medical University, Chongqing, 400038 China

**Keywords:** Human leukocyte antigens, Promiscuous T-cell epitopes, Supertype-specific epitopes

## Abstract

**Background:**

Promiscuous T-cell epitopes that can be presented by multiple human leukocyte antigens (HLAs) are prime targets for vaccine and immunotherapy development because they are effective in a high proportion of the human population. Although there are a number of epitope databases currently available online, the epitope data in these databases were annotated using specific MHC restrictions, and none of these databases was specifically designed for retrieving data on promiscuous epitopes.

**Description:**

HLAsupE is an integrated database of HLA supertype-specific epitopes (promiscuous T-cell epitopes in the context of HLA supertypes). The source data for the T-cell activities and HLA-binding capacities of peptides with a specific HLA restriction were extracted from public epitope databases. After a manual curation, these allele-specific data were integrated into supertype-specific datasets based on the defined supertypes and corresponding alleles. Each supertype-specific peptide in HLAsupE is annotated in terms of its cross-reactivity to HLA molecules within the same supertype. Promiscuous peptides that can be presented by multiple HLA molecules across multiple HLA supertypes were also included in this database. Several web-based tools are provided to access and download the data.

**Conclusions:**

HLAsupE is the first database of promiscuous T cell epitopes that is organized based on the HLA supertypes. The main advantage of this database is the ability to search for promiscuous T-cell epitopes based on the cross-reactivity to specific alleles or supertypes. HLAsupE will be a valuable resource for the development of epitope-based vaccines and immunotherapies with broad coverage of human population.

## Background

In the vertebrate immune system, short peptides derived from endogenous or exogenous antigens are presented by major histocompatibility complex (MHC) molecules on the surface of antigen presenting cells for recognition by T-cell receptors (TCRs). MHC-presented peptides that can trigger cell-mediated immune responses are termed T-cell epitopes and play a vital role in the development of epitope-based vaccines and immunotherapies against viral infections, tumors and autoimmune diseases [1–4]. However, human MHC (human leukocyte antigens, HLAs) genes exhibit a high level of polymorphism, and their distribution in the human population varies with ethnicity and region. Therefore, population coverage is a key question that should be considered during the development of epitope-based vaccines. Promiscuous T-cell epitopes that can be presented by multiple MHC molecules have great potential in the development of vaccines with wide population coverage, as fewer epitopes would be needed to cover a larger portion of specific populations [5, 6].

At present, a number of epitope databases, such as SYFPEITHI [7], MHCBN [8], AntiJen [9], and IEDB [10], have been constructed and reported. However, the data in these databases are annotated using specific MHC restrictions, and none of these databases was specifically designed for retrieving data on promiscuous epitopes. As a result, the retrieval of such data from these databases is indirect and often requires users to possess previous experience with sequence analysis. A more specific database would be of great interest for immunologists and vaccinologists who aim to develop novel vaccines with broad coverage of the human population.

Although more than 10,000 HLA alleles have been identified to date [11], most HLA molecules can be clustered into supertypes based on their overlapping peptide-binding specificities or the residue composition at their peptide-binding sites [12–15]. Peptides that bind to an HLA molecule with high affinity can also bind to multiple molecules within the same supertype [16]. These promiscuous epitopes in the context of HLA supertypes are defined as HLA supertype-specific epitopes. HLAsupE is an integrated database of HLA supertype-specific epitopes. The source data for the T-cell activities and HLA-binding capacities of peptides with a specific HLA restriction were extracted from public epitope databases. After a manual curation, these allele-specific data were integrated into supertype-specific datasets based on the defined supertypes and corresponding alleles. Each of the supertype-specific peptides in HLAsupE was annotated in terms of its cross-reactivity to HLA molecules within the same supertype. Promiscuous peptides that can be presented by multiple HLA molecules across multiple HLA supertypes were also included in this database. HLAsupE is the first database of promiscuous T cell epitopes that is organized based on the HLA supertypes. HLAsupE will be a valuable resource for the development of epitope-based vaccines and immunotherapies with broad coverage of human population.

## Construction and content

HLAsupE is a web-based server that combines a MySQL database management system and Perl programs with a dynamic web interface based on PHP.

### Data source and curation

Data on the T cell activity and HLA-binding capacity of peptides were mainly extracted from SYFPEITHI [7] and IEDB [10]. The overlapping peptide data extracted from different databases were first removed according to the PMID number. The T cell activities of peptides with specific HLA restrictions were classified into two groups, Positive (P) or Negative (N), based on the annotation in the source databases. The activity of a specific HLA-restricted peptide with multiple experimental results was defined using the relative number of positive and negative reports. The peptide was defined as "contradictory" (C) if the number of positive reports equaled the number of negative reports. The HLA-peptide binding capacity was determined by the quantitative binding affinity (IC50 or EC50). According to the conventional standard, peptides with a binding affinity stronger than 500 nM (IC50 < 500 nM) were classified as binders (Positive), and peptides with a weaker binding affinity (IC50 ≥ 500 nM) were defined as non-binders (Negative). Peptides without quantified binding affinities were classified based on their annotations in the source databases. The generated datasets, in which each specific HLA restricted peptide has a unique record, were used to define supertype-specific data.

### Generation of HLA supertype-specific datasets

Most of the known HLA class I and class II molecules can be clustered into supertypes. The HLA class I supertypes for the HLA-A and B loci used here were defined by Sidney et al [15], and the supertypes used for the HLA-C loci were based on the classification presented by Doytchinova [12]. The HLA class II supertypes used were consistent with Doytchinova’s definition [13]. Due to the relatively low number of HLA-DPA and DPB alleles with available peptide data, all available HLA-DPA and DPB alleles in this database were classified into one DP supertype. The supertypes and alleles with available peptide data can be found on the webpage of HLAsupE (http://www.immunoinformatics.net/HLAsupE/downloads/alleles.xlsx). The curated T cell activities and HLA-binding capacities of the peptide data were integrated into supertype-specific datasets based on HLA restriction and supertype. Each peptide in the supertype-specific datasets was annotated in terms of its cross-reactivity to HLA molecules within one supertype.

### Architecture of HLAsupE

**Fig. 1:**
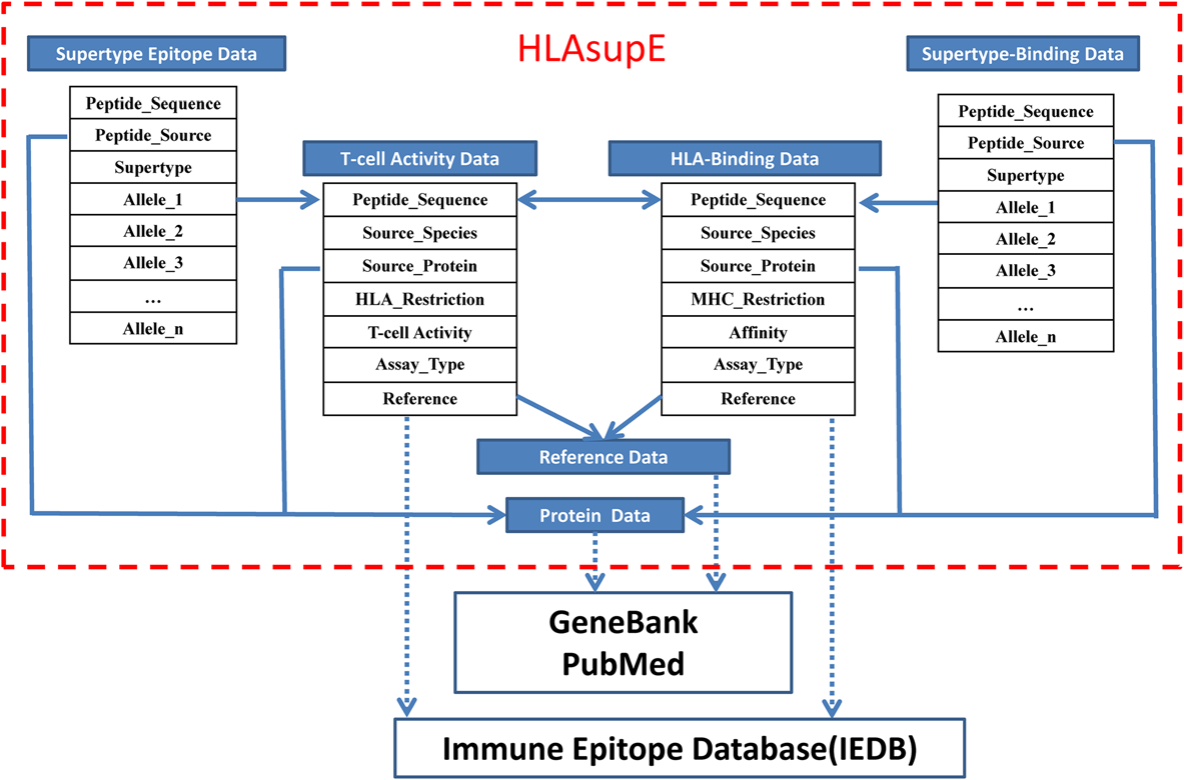
Architecture of HLAsupE

HLAsupE consists of six interrelated data blocks: (1) *HLA supertype-specific epitope* data: the restriction of promiscuous epitopes to HLA molecules within one supertype and basic information on the epitopes (e.g., sequence, source protein and organism); (2) *HLA supertype-specific binding peptide* data: the cross-binding abilities of peptides to HLA molecules within one supertype and basic information on the peptides; (3) *HLA-peptide binding* data: detailed information concerning the binding ability of a given peptide to a certain HLA molecule; and (4) *T-cell activity* data: the T-cell activity of a given HLA restricted peptide. The quantitative *HLA-peptide binding* data and the detailed *T-cell activity* data available in HLAsupE were mainly extracted from IEDB, and hyperlinks to the source data are provided. The *Source Protein* (5) and *Reference* data (6) are also imbedded in this database, and hyperlinks to GenBank and PubMed are provided. An overview of the database and the contents of the data blocks are schematically represented in Fig. 1.

### Statistics of HLAsupE

The latest version of the database maintains 17,889 unique records of HLA supertype-specific epitopes (SupEs) and 107,747 records of HLA supertype-specific binding peptides (SupBs). Non-redundant datasets on the detailed T-cell activity and HLA-binding capacity of specific HLA restricted peptides contain 31,793 and 196,510 records, respectively. Statistics based on HLA supertypes are shown in Table 1. The numbers of alleles with available peptide data in the SupE and SupB datasets are 195 and 204, respectively. The *Source Protein* dataset contains more than 14,000 proteins from approximately 1400 different source species and strains. The peptide datasets contained in HLAsupE can be freely downloaded at the download page (http://www.immunoinformatics.net/HLAsupE/download.htm).

**Table 1:** Statistical analysis of data included in HLAsupE

Label	Supertype	SupE	SupB	T-cell activity			HLA-binding	
				P	N	C	P	N
A	A1	1136	6839	108	2518	9	2817	10175
B	A2	4124	15625	1615	2465	182	13623	19297
C	A3	1904	9397	259	7519	108	9190	15557
D	A24	1599	4341	230	2526	16	1714	4280
E	B7	1224	6229	470	1730	79	3232	7488
F	B8	35	3434	31	4	--	1489	2402
G	B27	119	3952	106	30	2	1333	5492
H	B44	1256	8354	148	4254	5	6368	6642
I	B58	95	5151	98	4	2	2414	5466
J	B62	165	5224	80	82	3	2362	4385
K	C1	18	619	21	--	--	580	63
L	C4	48	1299	45	3	--	560	791
M	A01A03	8	2164	6	1	1	586	1578
N	A01A24	664	2145	27	636	1	858	1287
O	DP	173	1225	112	74	2	1115	3984
P	DQ1	337	1752	174	164	14	1005	2239
Q	DQ2	139	1630	140	34	2	507	1343
R	DQ3	332	1760	187	247	9	1113	2840
S	DQA	--	90	--	--	--	--	360
T	DR1	1604	10262	1373	510	33	8713	7902
U	DR3	620	3741	421	232	14	1577	5120
V	DR4	1353	5082	1104	446	29	4751	6827
W	DR5	549	4545	531	121	2	3970	6359
X	DR9	387	2887	335	57	2	2075	2681
	SUM	17889	107747	7621	23657	515	71952	124558
				31793			196510	

SupE and SupB: the statistics of unique records restored in the HLA supertype-specific epitope data block and the HLA supertype-specific binding peptide data block. The statistics on T-cell activity and HLA-binding capacity, which represent the number of HLA-peptide complexes with experimentally verified activity, were derived from non-redundant records. P, N and C represent positive, negative and contradictory records, respectively

## Utility

### Usage of HLAsupE

To facilitate the use of HLAsupE, we provide several online tools allowing users to search for and analyze HLA supertype-specific peptides. The following options are provided: (1) retrieving and browsing supertype-specific peptides by peptide sequence, supertype, cross-reactivity to specific alleles and source species; (2) mapping supertype-specific peptides onto a specific protein sequence; and (3) searching for mutant analogues of a specific peptide. These servers are easy to use, and a tutorial is also provided on the tutorial page: (http://www.immunoinformatics.net/HLAsupE/tutorial.htm).

**Fig. 2:**
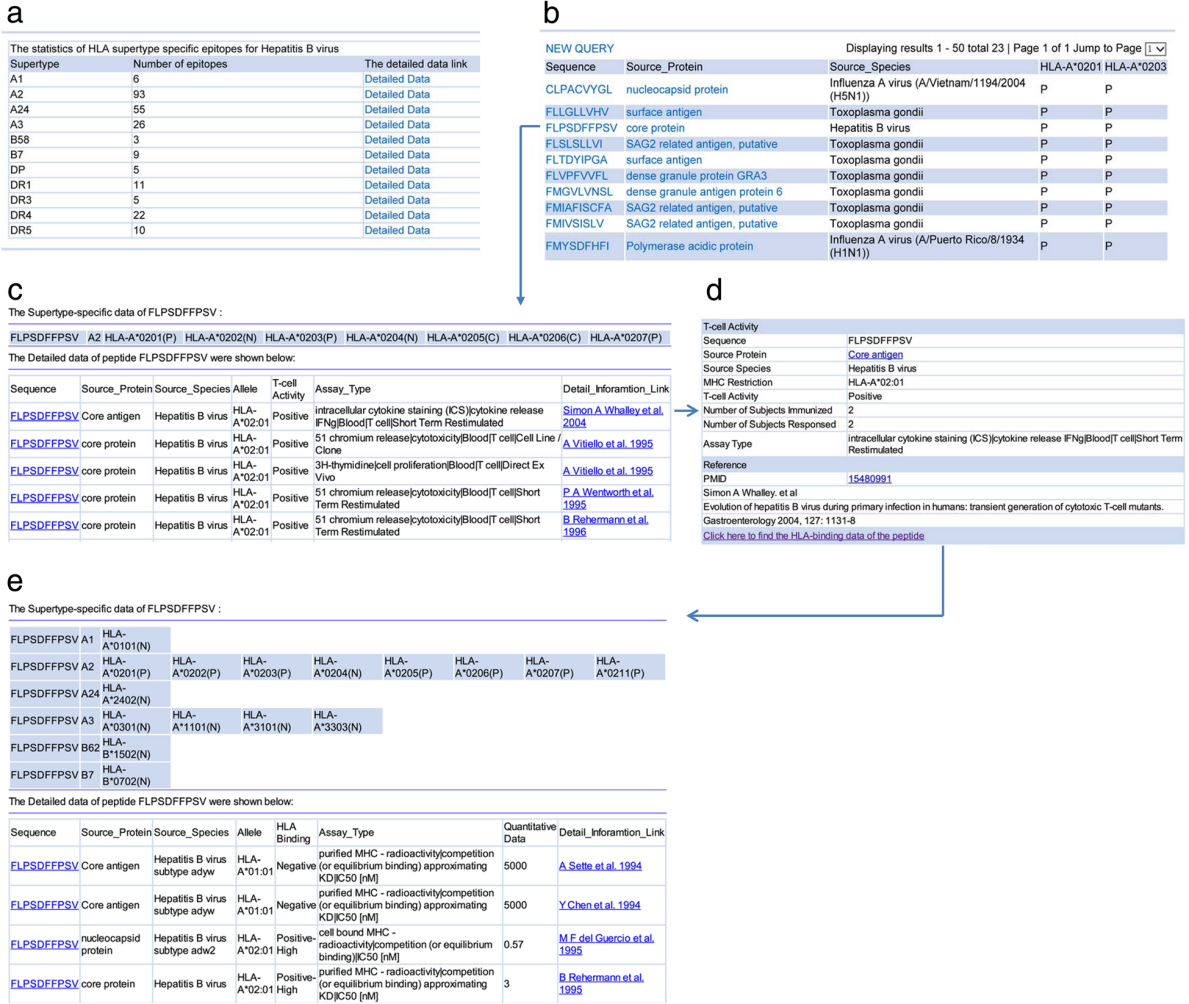
Screenshots of HLAsupE. **a** HLA supertype-specific epitopes browsed by source species (Hepatitis B virus); **b** HLA supertype-specific epitopes with two restrictions (HLA-A*0201(P), A*0203(P)); **c** Overview of the cross-reactivity of peptide “FLPSDFFPSV” to alleles of the same supertype followed by the experimental results obtained using different assay types or by different labs; **d** Detailed information of the first record in C; **e** Overview of the cross-binding ability of peptide “FLPSDFFPSV” to alleles of the same supertype and the experimental results obtained using different assay types or by different labs

We can use a query of HLA supertype-specific epitopes as an example to demonstrate the usage of HLAsupE. If a user chooses a specific source species, e.g., “Hepatitis B virus”, the statistics of epitopes related to HBV will be shown based on supertypes in tabular format (Fig. 2a). “Detailed Data” will give the curated T-cell activities of peptides presented by alleles of the same supertype in a similar format as in Fig. 2b. Figure 2b lists the peptides (sectional) that could be presented by HLA-A*02:01 and HLA-A*02:03 that share positive T cell activity for the restrictions of both alleles. The source protein and species of each peptide are also given in the results. The T-cell activities of each peptide listed in Fig. 2b are provided through a hyperlink and presented in the format depicted in Fig. 2c, which displays an overview of the cross-reactivity of the selected peptide to alleles of the same supertype followed by the experimental results obtained using different assay types or by different labs. The detailed data for each record listed in Fig. 2c are presented as shown in Fig. 2d. If a user is interested in the HLA-binding abilities of the selected peptide, the cross-binding abilities of the peptide to alleles can be obtained by clicking “*Click here to find the HLA-binding data of the peptide*” and are shown in the format presented in Fig. 2e, which presents an overview of the cross-binding ability to alleles of the same HLA supertype and the experimental results obtained using different assay types or by different labs. The query method for HLA supertype-specific binding peptides is the same as that for supertype-specific epitopes.

### Promiscuous peptides in the context of different supertypes

In addition to HLA supertype-specific peptides, HLAsupE contains a large number of promiscuous peptides that can be presented by multiple HLA molecules within different supertypes (Epitopes: 630, Binders: 5,166). The collection of these promiscuous T-cell epitopes provides additional evidence for understanding HLA function and the development of epitope-based vaccines. The statistics on promiscuous peptides across every two supertypes are listed in Table 2. Promiscuous binding mainly occurs between supertypes of the same HLA class (class I or II), but some peptides can be presented by multiple alleles across HLA classes. To facilitate the use of these promiscuous peptides, we also developed query tools for promiscuous T-cell epitopes and for promiscuous binding peptides (http://www.immunoinformatics.net/HLAsupE/cross.html). These data can be output based on the source species or/and the supertype selected by the user.

**Table 2:** Statistical analysis of promiscuous peptides across every two supertypes

	A	B	C	D	E	F	G	H	I	J	K	L	M	N	O	P	Q	R	T	U	V	W	X
A	--	193	152	81	131	28	98	22	207	295	32	24	319	61									
B	9	--	119	68	114	71	100	45	115	156	31	23	29	37		1				1			
C	3	4	--	20	62	7	57	16	96	112	34	16	47	112					5	4	4	2	4
D	5	16	3	--	44	8	10	12	48	58	8	6	101	7									
E	3	15	3	11	--	71	58	20	134	227	21	8	14	16									
F		3			1	--	65	6	19	79	7	6		25					1		1	1	1
G		3				1	--	44	34	157	35	20	4	46									
H	1	2			1	1		--	26	33	3	10	9							1			
I		1	1	2	2				--	192	20	8	25	27									
J	1	2		3	3					--	32	8	44	44					1		1		
K		3			2		2				--	20	9	1									
L	4			5					1		1	--	2	5									
M	10	1		2									--	4									
N	2	1	3		1					1				--									
O			1									2			--	192	99	200	310	168	270	285	265
P															2	--	104	336	341	177	292	320	292
Q															1	3	--	138	127	92	134	108	100
R											1				1	43	4	--	476	234	402	403	380
T		19	2	10	5	1									9	17		26	--	782	1714	1550	1310
U		9		4	1										6	7	1	9	53	--	746	724	583
V		10	1	9	3										5	18	1	12	171	51	--	1361	1088
W		6		1	1										7	8		13	93	38	71	--	1089
X		2		2											5	10		1	87	7	32	34	--

Promiscuous peptides that can be presented by multiple HLA molecules within different supertypes were collected, and a statistical analysis of these promiscuous data across every two supertypes was performed. Statistics on promiscuous epitopes are shown in the lower triangle, and statistics on promiscuous binding peptides are shown in the upper triangle. The values in the table indicate the numbers of peptides that can be presented by HLA molecules within the two supertypes indicated in the row and column headers. The supertype names are presented in capital letters according to the labels in Table 1

## Discussion

HLAsupE is a database of promiscuous T cell epitopes that is organized based on HLA supertypes. Although promiscuous binding has been considered a hallmark of HLA class II restricted peptides [17–19] and the promiscuous recognition of CTL epitopes in the context of unrelated HLA class I molecules has been reported and investigated [20], supertype-based cross-binding remains predominant in promiscuous data. Moreover, the supertypes of HLA molecules have been well defined [12, 13, 15], which make it possible to integrate the promiscuous peptides or epitopes based on HLA supertypes.

The data in HLAsupE were extracted from SYFPEITHI [7] and IEDB [10]. The widely known database SYFPEITHI [7] contains only positive data and lacks quantitative descriptions of these peptide data and negative reactive peptides. The quantitative HLA-peptide binding data and the detailed T-cell activity data available in HLAsupE were mainly extracted from IEDB. IEDB is the largest database of immune epitopes and covers almost all peptide data in the other known epitope databases. The experimental data on the T-cell activity and MHC-binding capacity of peptides included in IEDB have been detected using various assay methods or submitted by different laboratories. Therefore, the redundancy of data for specific MHC-restricted peptides is inevitable. In HLAsupE, the T-cell activity and MHC-binding capacity of each HLA-restricted peptide were curated based on the available experimental data, and supertype-specific data were generated using these curated data such that each HLA-restricted peptide has a unique record. Thus, the data included in the *Supertype Epitope* and *Supertype-binding* data blocks (Fig. 1) are non-redundant. To maintain the integrity of the data, the *T-cell activity* and *HLA-peptide binding* data blocks contain all the data extracted from the source databases, which are highly redundant because of the overlap of peptide data in different databases and the inherent redundancy of the data in the source database. In HLAsupE, a hyperlink to the publication or source database is provided for each record of the detailed information of specific-allele-restricted peptides to ensure data traceability, which should help the user to further inspect a specific epitope, particularly when encountering records with contradictory results.

As a database of promiscuous epitopes, the main advantage of HLAsupE with respect to the existing databases of T-cell epitopes is that each epitope in HLAsupE was annotated with its cross-reactivity to HLA molecules within one supertype (HLA supertype-specific data blocks). As a result, users can instantly retrieve the promiscuous epitopes with multiple selected HLA restrictions. The query tools in HLAsupE now allow users to define the promiscuous binding ability using five different HLA molecules within one supertype. Moreover, HLAsupE can also be used for the query of promiscuous peptides with different binding affinities to multiple HLA molecules, e.g., peptides that can bind to HLA-A*0201and A*0202 but not A*0203 [A*0201(+), A*0202(+), A*0203(-)], which would be useful for the analysis of allele-specific recognition patterns. In addition to HLA supertype-specific peptides, HLAsupE also has a collection of a large number of promiscuous peptides that can be presented by multiple HLA molecules within different supertypes and provide corresponding query tools for these promiscuous data.

There are large amounts of data on peptides presented by serological HLA molecules (such as HLA-A2, B51 B7 and DR1) included in SYFPEITHI and IEDB. However, the HLA supertypes in HLAsupE were defined based on the published data [12, 13, 15], and the HLA molecules encoded by HLA alleles (genotype) were used in the identification of the HLA supertypes. Therefore, the peptides presented by serological proteins were difficult to integrate into the supertype-specific dataset with an exact allele restriction. These data were only maintained in the *T-cell activity* and *HLA-peptide binding* data blocks; as a result, these peptide data can be displayed when a user inspects the detailed activity of a specific peptide, but a direct query of these peptides is currently unavailable in HLAsupE.

The MHC restriction and population coverage are key questions in the development of epitope-based vaccines that contain at least two antigenic epitopes: a Th-epitope and an epitope that will either induce specific B-cell or CTL responses [4]. Promiscuous T-cell epitopes have great potential in the development of vaccines with wide population coverage. However, the distribution of HLA genes in the human population varies with ethnicity and region. The frequency of alleles in a specific population should be taken into account. At present, Allele Frequency Net Database (AFND) [21] and Population Coverage tool [22] have been built to address this issue. By combining with these tools, our database should be more useful in practice.

We will continue to update our database by extracting and curating HLA-restricted peptide data from all available epitope databases and published literatures. To make HLAsupE an even more powerful resource, we will further improve the functionality and architecture of HLAsupE to facilitate the use and analysis of promiscuous peptides.

## Conclusions

HLAsupE is the first database of promiscuous T cell epitopes and was constructed by integrating allele-specific data into supertype-specific data. The main advantage of this database server is the ability to search for promiscuous epitopes or binding peptides based on cross-reactivity to specific alleles or supertypes. This database is a valuable resource for the development of epitope-based vaccines with broad coverage of the human population and to obtaining a further understanding of the cellular immune response.

## Abbreviations

HLA, human leukocyte antigen; MHC, major histocompatibility complex; SupB, supertype-specific binding peptide; SupE, supertype-specific epitope; TCR, T-cell receptor
